# Shannon Entropy in Uncertainty Quantification for the Physical Effective Parameter Computations of Some Nanofluids

**DOI:** 10.3390/nano15030250

**Published:** 2025-02-06

**Authors:** Marcin Kamiński, Rafał Leszek Ossowski

**Affiliations:** Faculty of Civil Engineering, Architecture and Environmental Engineering, Lodz University of Technology, 93-590 Łódź, Poland; rafal.ossowski@gmail.com

**Keywords:** nanofluids, Shannon entropy, effective properties, Monte-Carlo simulation, homogenization

## Abstract

The main aim of this study is probabilistic computer simulation of the effective physical parameters of fluids containing nanoparticles. A deterministic model following the rule of mixtures and some semi-empirical formulas are employed to calculate effective density, heat conductivity, heat capacity, as well as viscosity for the given nanofluid. This models is randomized here using the Monte-Carlo simulation apparatus for estimation of the Shannon entropy of all these physical parameters, which is the crucial novelty of this study. The volume fraction of the nanoparticles is assumed for this purpose as the Gaussian uncertainty source with the given first two moments. The basic probabilistic characteristics of the nanofluids’ homogenized parameters have also been determined here for some validation of Shannon entropy variations in addition to the statistical disorder of the nanoparticle fraction. These research findings contribute to advancing nanofluidic and microfluidic research, offering robust tools for uncertainty analysis and enhancing the reliability of physical parameter predictions in applications requiring high numerical and/or experimental precision.

## 1. Introduction

**Figure 1:**
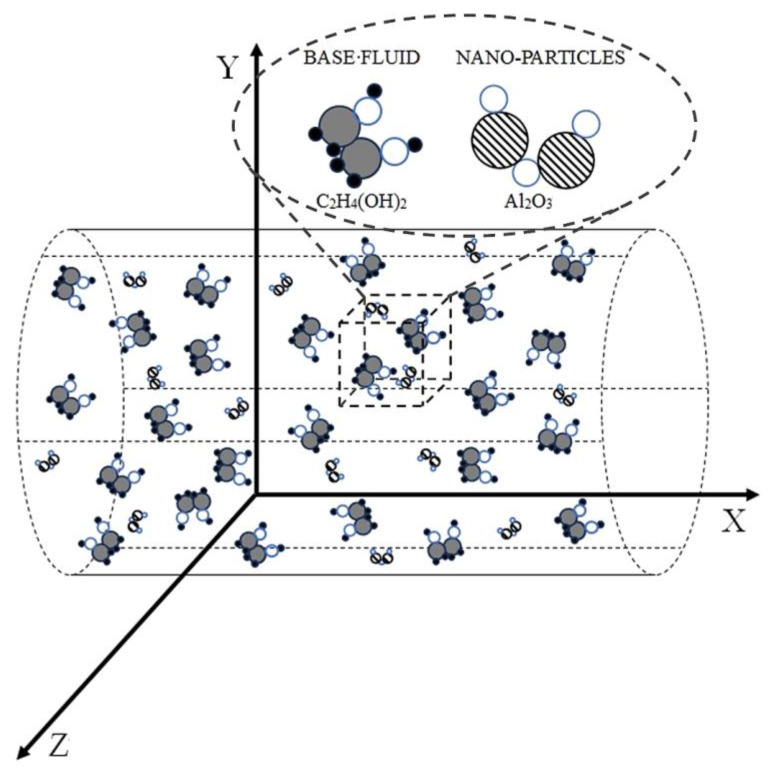
The general structure of the nanofluid.

Determination of the effective parameters of composites composed of coupled solid components, as well as multiphase mixtures, is a fundamental topic within homogenization theory [1]. This approach has a well-established history and encompasses a range of mathematical, computational, and semi-experimental methods, including applications in nanofluidics [2]. As known from the literature, all these homogenization models could be divided into a direct algebraic approximation of the effective parameters like a rule of mixtures and their improvements, upper and lower bound estimates [3] as well as a variety of numerical implicit methods generally based upon some physical equivalence and machine learning apparatus [4]. This equivalence most frequently includes equity of deformation energy of the real and homogenized medium and may be provided using different boundary conditions imposed on the Representative Volume Element (RVE). The concept of nanofluids represents a significant advancement in the targeted modification, enhancement, and design of the physical properties of fluid-based mixtures [5]. These mixtures are engineered to exhibit optimized density, thermal conductivity [6,7], viscosity, and heat capacity by incorporating nanoparticles into the base fluid. Such precise control over these parameters has far-reaching implications, particularly in the development of advanced cooling systems, energy-efficient thermal management solutions, and enhanced material processing techniques. Nanofluids provide a platform for tailoring the thermal and rheological behavior of fluids to meet specific industrial and technological demands, thereby broadening their applications in sectors such as machining [8], electronics, transportation, and renewable energy systems [9]. A nanofluid is a liquid substance composed of a liquid base with an admixture of solids in the form of nanoparticles; the size of the molecules is smaller than 100 nm, where the content usually does not exceed 4% of the total volume. The base of such a nanofluid may be any organic liquid or water [10,11], while the most commonly used molecules are metals (Au, Cu, C), oxides (CuO, Al_2_O_3_ [12], SiO_2_, TiO_2_ [13], ZrO_2_ [14]), and carbon nanotubes. The process of enriching fluids with solid particles to alter their properties dates back over a century and has evolved significantly with advancements in materials science. Early studies focused on suspensions of micron-sized particles to enhance the density and viscosity of fluids for specific industrial applications, such as drilling muds and concrete slurries. For example, the use of suspended solids to modify fluid flow and improve performance in hydraulic systems was documented as early as the early twentieth century. Modern advancements have transitioned from using micron-sized particles to nanoscale particles, which offer superior control over fluid properties due to their high surface area-to-volume ratio and unique thermal and mechanical behaviors [15]. The inventor of this idea was most probably J.C. Maxwell, but only the development of electron and tunneling microscopy in the second half of the previous century allowed for full experimental verification of theoretical predictions [16,17]. Calculation of the effective material and physical characteristics of the nanofluids still remains important and a challenging research problem due to the fact that such characteristics avoid multiscale and multiphase computational modeling of such media and enables relatively faster solutions of the flow problems described using the Navier–Stokes equations [18,19]. The main shortcoming of many of the existing models is that statistical scattering of nanofluids’ properties is usually postponed even if some experimentation is presented; the same concerns an impact of this scattering on or within their effective characteristics (Figure 1).

Taking the above into account, the deterministic philosophy presented in [2] is extended towards a probabilistic model, where the basic random parameter is the volumetric ratio of the nanoparticles. This is proposed to make the model consistent with the needs of uncertainty quantification, propagation, and also with the stochastic reliability models; this need is also driven by some engineering applications of this type of fluids [8,20]. Further, it is almost impossible to insert the very specific and precisely measured, deterministic amount of the nanoparticles and to mix all of them with a large amount of the liquid base, which leads to relatively large variations (frequently uncertain) in the effective properties of nanofluids in addition to small changes in input parameters. The observed nonlinear entropy fluctuations indicate critical thresholds at which minor variations in input uncertainty result in disproportionately large changes in the effective properties. These thresholds may be associated with physical phenomena such as percolation transitions, nanoparticle clustering, or modifications in heat transfer mechanisms.

The homogenization approach for calculation of effective physical properties of the nanofluid is presented first [21], and it is extended next using statistical Monte-Carlo methodology [22] towards statistical estimation of the first two probabilistic moments of the effective parameters of the nanofluids. Additionally, Shannon entropy is computed using numerical recovery and specific further processing of the resulting histograms of effective parameters separately. Using additional partition of such a histogram, it was possible to implement the Shannon entropy [23] scheme in the system MAPLE 2024 [24]. This numerical tool was engaged in the calculation of these basic statistics for effective physical parameter uncertainties of the glycol ethylene fluid base enriched with the alumina particles. A final comparison of the expectations and coefficients of variation with the Shannon entropy fluctuations obtained for different mean values of the volumetric ratio of these nanoparticles shows uncertainty propagation in nanofluids’ properties. It also enables a validation of the Shannon entropy apparatus in such statistical analysis, which has been applied before in some nonlinear problems of solid mechanics [25]. Let us note that the uncertainty analysis was based here upon the assumption that the input statistical scattering is in nanoparticle volume fraction having Gaussian distribution, while Weibull probability distribution was also employed [26]. This choice is driven by the Maximum Entropy Principle, where Gaussian uncertainty causes largest uncertainties in physical systems. A new element of this work is the uncertainty quantification of the effective parameters in nanofluids using a single parameter—Shannon entropy—instead of multiple graphs of probabilistic moments as applied before. It remarkably simplifies any stochastic analyses in the area of homogenization in nanofluidics, whereas applications of any probabilistic entropy in stochastic computational dynamics still remain very scarce.

## 2. Basic Equations for the Uncertainty Quantification in Nanofluid Parameters

Let us consider fundamental Navier–Stokes flow equations in the given computational domain Ω for the unknown fluid state functions, i.e., velocities *v_i_* = *v_i_(**x**)*, pressure *p = p(**x**)*, and temperature *θ = θ(**x**)* in their following classical formulation [19,27]:

(1) $$\rho \left(\frac{\partial v_{i}}{\partial t}+v_{i,j}v_{j}\right)=\sigma_{ij,j}+\tilde{f_{i}},$$

(2) $$v_{i,i}=0,$$

(3) $$\sigma_{ij}=-p\delta_{ij}+2\mu \varepsilon_{ij},$$

(4) $$\rho c\left(\frac{\partial \theta }{\partial t}+\theta_{i}v_{i}\right)={\left(k\theta_{i}\right)}_{i}+{\tilde{q}}_{i},$$

where strain tensor is described as

(5) $$\varepsilon_{ij}=\frac{1}{2}\left(v_{i,j}+v_{j,i}\right)=\frac{1}{2}\;\left(\frac{\partial v_{i}}{\partial x_{j}}+\frac{\partial v_{j}}{\partial x_{i}}\right),\;\;\;i=1,2,3.$$

The tensor $\sigma_{ij}$ represents here the stress state in the given fluid, whose viscosity is denoted by *μ*, its heat conductivity by *k*, heat capacity as *c*, and mass density by *ρ*. These equations are relevant to macroscopically homogeneous single-phase fluids but can also be applied to heterogeneous systems as long as they exhibit similar behavior on a macroscopic scale. In the context of nanofluids, such an approach allows for modeling systems where nanoparticles are unevenly distributed, yet their impact on thermal or rheological properties is significant at the macroscopic level. The following general boundary conditions are applicable.

(6) $$v_{i}={\hat{v}}_{i};x\in \partial \Omega_{v},\;\sigma_{ij}n_{j}={\hat{f}}_{i};x\in \partial \Omega_{\sigma },\;\theta =\hat{\theta };x\in \partial \Omega_{\Theta }\;k\frac{\partial \theta }{\partial x}=\hat{q};x\in \partial \Omega_{q}.$$

Let us assume further that the computational domain for these NS equations is filled with the nanofluid, which means that this is a mixture of the homogeneous liquid, which is a continuous basis (*f*) and a discontinuous solid in the form of molecules (*p*), and its flow at the macroscale is similar in a physical sense to a homogeneous fluid having effective parameters calculated from the corresponding characteristics of the original fluid and the nanoparticles. Therefore, instead of Equations (1)–(4), one solves the equivalent NS equations system with the homogenized coefficients (with the subscripts ‘*nf*’) as

(7) $$\rho_{nf}\left(\frac{\partial v_{i}}{\partial t}+v_{i,j}v_{j}\right)=\sigma_{ij,j}+\tilde{f_{i}},$$

(8) $$v_{i,i}=0,$$

(9) $$\sigma_{ij}=-p\delta_{ij}+2\mu_{nf}\varepsilon_{ij},$$

(10) $$\rho_{nf}c_{nf}\left(\frac{\partial \theta }{\partial t}+\theta_{i}v_{i}\right)={\left(k_{nf}\theta_{i}\right)}_{i}+{\tilde{q}}_{i},$$

with the same boundary conditions as before except for the heat flux, where the fluid heat conductivity is replaced with the effective one. The effective density, which opens a collection of the effective characteristics, is calculated according to the simple mixtures rule, where *φ* serves as the volume ratio of the nanoparticles; this holds similarly to heterogeneous solids models [1,2]:

(11) $$\rho_{nf}={\left(\frac{m}{V}\right)}_{nf}=\frac{m_{f}+m_{p}}{V_{f}+V_{p}}=\frac{\rho_{f}V_{f}+\rho_{p}V_{p}}{V_{f}+V_{p}}=(1-\phi )\rho_{f}+\phi \rho_{p}$$

For typical nanofluids, where the percentage of fixed molecules does not exceed 1% by volume of the substance, the observed change in density does not usually exceed 5%. Then, the effective heat capacity is calculated in quite a similar way, i.e.,

(12) $$\begin{array}{l} {\left(\rho c_{p}\right)}_{nf}=\rho_{nf}{\left(\frac{Q}{m\Delta T}\right)}_{nf}=\rho_{nf}\frac{Q_{f}+Q_{P}}{\left(m_{f}+m_{p}\right)\Delta T}=\rho_{nf}\frac{{\left(mc_{P}\right)}_{f}\Delta T+{\left(mc_{P}\right)}_{p}\Delta T}{\left(m_{m}+m_{p}\right)\Delta T}=\\ =\rho_{nf}\frac{{\left(\rho c_{P}\right)}_{f}V_{f}+{\left(\rho c_{P}\right)}_{p}V_{p}}{\rho_{f}V_{f}+\rho_{p}V_{p}}=\left(1-\phi \right){\left(\rho c_{P}\right)}_{f}+\phi {\left(\rho c_{P}\right)}_{p} \end{array}$$

so that the following holds true:

(13) $$c_{nf}=\frac{\left(1-\phi \right){\left(\rho c_{P}\right)}_{f}+\phi {\left(\rho c_{P}\right)}_{p}}{\left(1-\phi \right)\rho_{f}+\phi \rho_{p}}=\frac{\left(1-\phi \right){\left(\rho c_{P}\right)}_{f}+\phi {\left(\rho c_{P}\right)}_{p}}{\rho_{nf}}$$

Using Equation (13), one may predict the changes in the specific heat value assuming a uniform dispersion of molecules in a liquid. Effective thermal conductivity has a more complex determination. In general nanofluids, it depends upon eight key parameters:

(a) The volume concentration of molecules, where an increase in the concentration of the molecules usually increases the thermal conductivity values for the nanofluid.
(b) Particle size, where in most cases, an increase in their diameter yields the additional increase in nanofluid conductivity; however, some specific experimental works demonstrate an inverse relation.
(c) The shape of nano molecules, where elongated shapes increase the effective thermal conductivity concerning spherical particles.
(d) Material properties of particles.
(e) Temperature, whose increase yields an additional increase in effective conductivity,
(f) Material properties of the liquid base (an increase in thermal conductivity coefficient of the nanofluid in addition to the based fluid).
(g) Extra components, which are added to the liquid to maintain the nanoparticles in suspension and prevent them from clumping; enrichment of the base fluid increases the coefficient of thermal conductivity for the nanofluids.
(h) Acidity, whose increase is proportional to an increase in the overall conductivity coefficient.

It should be noted that the chemical composition of a given nanofluid may significantly influence the sensitivity of the physical parameters under consideration. While modeling the effective thermal conductivity coefficient, several algebraic approximations can be applied. Among these, the rule of mixtures is often the initial choice; however, it is widely acknowledged as insufficiently accurate for capturing the complex interactions inherent in nanofluid systems. Alternative approaches, incorporating particle–fluid interactions and nonlinear effects, have demonstrated superior performance in accurately predicting thermal behavior across various experimental setups.

(14) $$k_{nf}=(1-\phi )k_{f}+\phi k_{p}$$

Alternatively, one may employ the model proposed by Maxwell based on the potential theory, which treats nanoparticles as homogeneous spheres, which does not interact with the other spheres, and all of them are embedded into the homogeneous viscous Newtonian fluid with no gas bubbles [2]. The following holds:

(15) $$\frac{k_{nf}}{k_{f}}=1+\frac{3\left(\frac{k_{p}}{k_{f}}-1\right)\phi }{\left(\frac{k_{p}}{k_{f}}+2\right)-\left(\frac{k_{p}}{k_{f}}-1\right)\phi }$$

for complex models, which may be an example of a model including the Brownian motion

(16) knfkf=1+ARemPr0.333Φ1+2kpkf+2Φ1+kpkf1+2kpkf+ϕ1+kpkf

The effective viscosity determination looks similar to the case of thermal conductivity, the appearance of the nanoparticles in a liquid base should result in viscosity increase in the nanofluids. Change in viscosity depending on the content of the nanoparticles in a liquid base can be predicted by the Einstein equation as

(17) $$\mu_{nf}=\left(1+2.5\phi \right)\mu_{f}\;\mathrm{gdy}\;\phi <0.05$$

This has some limitations with respect to the relatively small values of the volume fraction *φ*. Alternatively, one considers the Brinkman model, which is less sensitive to this volumetric ratio:

(18) $$\mu_{nf}=\frac{\mu_{f}}{{\left(1-\phi \right)}^{2.5}}$$

Further, we consider the volume fraction *φ* of the nanoparticles as the input uncertainty source, whose probability distribution causing the largest uncertainty in effective parameters according to the Maximum Entropy Principle should be the Gaussian one. Such a computer analysis is carried out in the following steps: (1) generation of a population of the volume fraction according to its statistical parameters (expected value and standard deviation); (2) sequential calculation of the effective parameters; and (3) statistical estimation of the first two probabilistic moments of these parameters [22]. However, it is known that uncertainty analysis based upon probabilistic moments and coefficients has some limitations and may be biased, for instance, by estimation numerical errors; therefore, a concept of Shannon probabilistic entropy *h* was additionally employed [23,25]. Its basic formula for the given statistical function *f* = *f*(*φ*) has been proposed in the literature as follows:

(19) $$h\left(f\left(\varphi \right)\right)=-\sum_{i=1}^{n}p_{i}\left(f\left(\varphi \right)\right)\ln\left(p_{i}\left(f\left(\varphi \right)\right)\right)\;$$

where *n* stands for the number of possible different states of this system. Since the coefficient of variation (CoV) is dominantly used in stochastic computational mechanics to discuss uncertainty importance and propagation in the given engineering problem, a comparison of Shannon entropy fluctuations with analogous changes in the CoV is investigated here.

The computer simulation of the effective physical characteristics, their probabilistic moments and Shannon entropy in the presence of some input uncertainty was programmed in the computer algebra system MAPLE 2024. This system was selected due to its Statistics library, its simplicity of statistical functions programming, its efficacy and satisfactory speed in probabilistic analyses programming and performance as well as nice and easy visualization of the resulting probabilistic characteristics. The statistical numerical simulation prepared for the needs of this study was based upon the traditional Monte-Carlo scheme and consisted of the following steps:

(i) Initial generation of random population for the given input parameter(s) of the nanofluid;
(ii) Sequential recalculation of a few populations of its effective physical characteristics;
(iii) Usage of statistical estimators for up to the fourth-order probabilistic characteristics of these effective parameters;
(iv) Creation of the histogram of the probability distributions for effective characteristics;
(v) Partition of this histogram into a few subintervals and final calculation of Shannon entropy.

The entire numerical simulation is parametrized with a few inputs and these are as follows: (i) input coefficient of variation in the given uncertainty source (whose impact has been demonstrated in all the resulting graphs); (ii) total number of random trials in Monte-Carlo scheme, whose impact was studied in the literature many times; and (iii) a number of subintervals proposed for the PDF partition at the last phase, which were adopted after [25]. Some additional internal parameters of symbolic computations precision like the number of digits may also have some importance in this analysis.

## 3. Numerical Results

**Figure 2:**
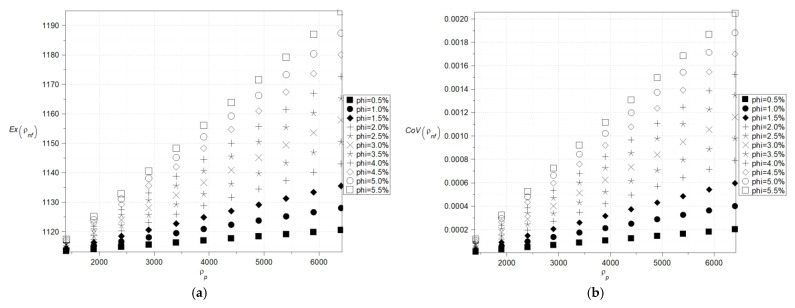
Expected values (**a**) and coefficients of variation (**b**) of the effective density (the density of the particles varies (1400–6400) kg/m^3^).

**Figure 3:**
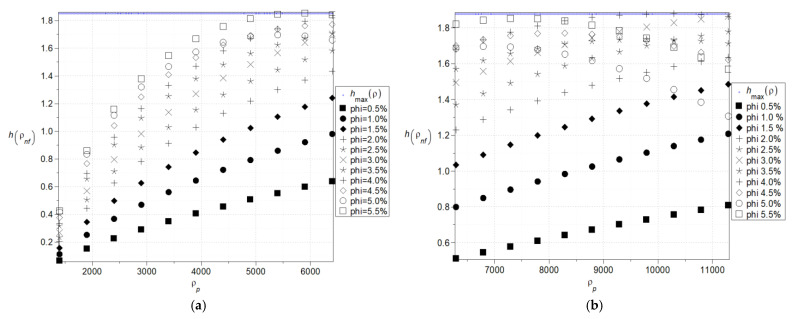
Shannon entropies for the effective density of the nanofluids with Al_2_O_3_ (**a**) and Cu (**b**) (the density of the particles varies (1400–11,400) kg/m^3^).

The main goal of the numerical results was to check whether uncertainty quantification in effective characteristics of the nanofluids can be performed using a single statistical disorder function (Shannon entropy) instead of a widely applicable multiple parametric study (expected values and standard deviations, for instance). The computer simulation enabling such a contrast was performed using the computer algebra system MAPLE 2024. However, analogous numerical calculations can be carried out with other available tools such as MATLAB R2024b, Mathematica 13.3, or MathCAD 10, offering flexibility in computational approaches depending on the specific requirements of the analysis. The computer algebra system MAPLE 2024 is well known for its powerful symbolic computation abilities and is widely employed in solving differential equations, simplifying algebraic expressions, and conducting dimensional analysis [24,25]. In all numerical experiments, the volume fraction, φ, is modeled as a Gaussian random variable characterized by its first two moments. A typical variation range for the volume fractions *φ* for the fluids with the nanoparticles is between 0.01 and 0.04 so the range 0.005–0.055 was initially preselected. The fluid base was chosen in the ethyl glycol C_2_H_4_(OH)_2_ with the following physical parameters: d = 1113 kg/m^3^, mi = 0.018396 Pa·s, C = 2382 J/kg·K, k = 0.25 W/m·K. But for the needs of sensitivity verification, it was additionally accepted that C = {1900, …, 2700} J/kg·K. Nanoparticles’ characteristics were adopted with the values typical for the alumina Al_2_O_3_, which means d = 3900 kg/m^3^, k = 29.5 W/m·K and as a reference point for determining the direction and magnitude of entropy changes, copper Cu (d = 8960 kg/m^3^, k = 400 W/m·K) was chosen, considering its practical applications [12,28]. The Monte-Carlo simulation as well as statistical estimation contained in the *Statistics* library were engaged in the computer program, where the total number of random trials for all simulations was fixed as equal to 200,000. The standard deviation of the fluid volumetric ratio was predefined as 5% of the corresponding expected value in all simulations. The results in the form of expected values, coefficients of variations as well as Shannon entropy of the effective nanofluid density are presented in Figure 2a,b and Figure 3 as the functions of both the particles’ density as well as their volume fraction. There is no doubt that the key parameter here is the volume fraction of the particles, and even a small increase in this leads to a rapid increase in the resulting density statistical parameters. The expected values and coefficients of variation, which were determined based on the model outlined in [22], and changes concerning the particle density are also remarkable and linear, whereas Shannon entropy fluctuations are nonlinear (Figure 3a,b), which is even more pronounced in the case of copper nanoparticles (Figure 3b). Additionally, they converge to some extreme value obtained at the upper end at the horizontal axis but for larger volumetric ratios only, which is also clearly observed for nanoparticles with higher density (Figure 3b). The trends observed for lower volume fractions in both cases continue to exhibit an increasing tendency in this region. This phenomenon may be attributed to the ordering of nanoparticles upon exceeding a certain volume fraction threshold—around 4%—which becomes more pronounced when there is a significant difference in relative density between the base fluid and the nanoparticles (Figure 3b). The significantly higher Shannon entropy observed for Cu nanoparticles compared to Al_2_O_3_ nanoparticles at the same *φ* parameter value (Figure 3a,b) can be attributed to Brownian effects. The size of Cu nanoparticles is approximately 45 nm, whereas Al_2_O_3_ nanoparticles range between 0.5 and 1.5 µm. In the initial stage of homogenization, Cu nanoparticles are more susceptible to thermal motion induced by the base fluid than Al_2_O_3_ particles. Since the Shannon entropy is proportional to the standard deviation for the given population, it is clear that the extreme uncertainty would be obtained for the extreme value expectation of the particle density and their extreme volumetric ratio expectation.

**Figure 4:**
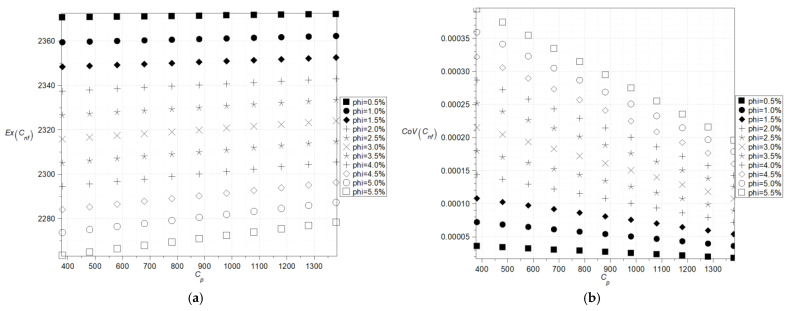
Expected values (**a**) and coefficients of variation (**b**) of the effective specific heat capacity.

**Figure 5:**
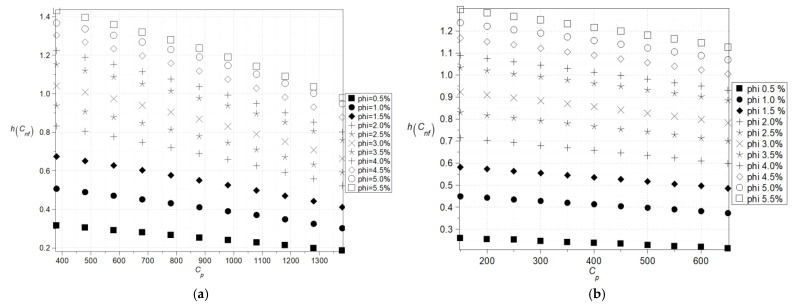
Shannon entropies for the effective specific heat capacity of the nanofluids with Al_2_O_3_ (**a**) and Cu (**b**) where the specific heat of the particles varies (380–1380) J/kg·K (**a**) and (160–640) J/kg·K (**b**).

The next two figures, cf. Figure 4 and Figure 5, show expectations (Figure 4a) and coefficients of variation (Figure 4b) as well as Shannon entropies of the effective specific heat capacity of the nanofluid with Al_2_O_3_ (Figure 5a) where the specific heat of the particles varies (380–1380) J/kg·K and with Cu (Figure 5b) where the specific heat of the particles varies (160–640) J/kg·K and volumetric ration of the nanoparticles does not exceed 5%. Now, the largest expectations of the effective heat capacity of the nanofluid are obtained with the smallest concentration of the nanoparticles, whereas minimum expectations are obtained for their largest amount. This expectation of effective capacity is almost insensitive to the input particles’ heat capacity for their smaller volume fractions in the given fluid base and linearly increases together with the particle’s heat capacity in the opposite situation.

Coefficients of variation as well as Shannon entropies decrease while increasing the input mean value of the particle’s heat capacity, and this inverse proportionality has an almost linear character. The largest values in both cases are obtained with the largest volumetric ratio of the particles and the smallest mean value of the particle’s heat capacity. It can be observed that, within the analyzed range, an increase in the average heat capacity and the volumetric fraction of particles leads to a decrease in entropy, which is clearly noticeable in (Figure 5a). This phenomenon may be related to the emergence of nonlinearities at critical values of the *φ* coefficient from the perspective of nanofluid theory or to physical processes such as aggregation or percolation.

In the case of poorly dispersed Al_2_O_3_ nanoparticles, molecular aggregation may occur, leading to a higher Shannon entropy compared to the corresponding situation for Cu nanoparticles (Figure 5a,b). This, in turn, can result in local fluctuations in the effective specific heat capacity of the nanofluid. This result is expected because the particle fraction is the uncertainty source and increasing its heat capacity mean value reduces the impact of the Gaussian volume fraction *φ*. Nevertheless, the coefficient of variation and Shannon entropy exhibit similar variations with almost constant expectations; probabilistic entropy has slightly smaller values than the effective nanofluid density in Figure 3.

**Figure 6:**
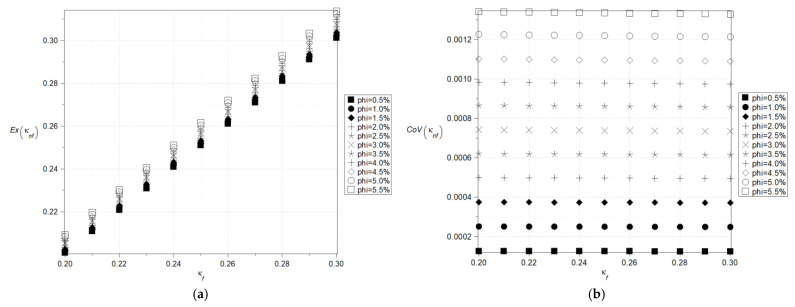
Expected values (**a**) and coefficients of variation (**b**) of the effective thermal conductivity.

**Figure 7:**
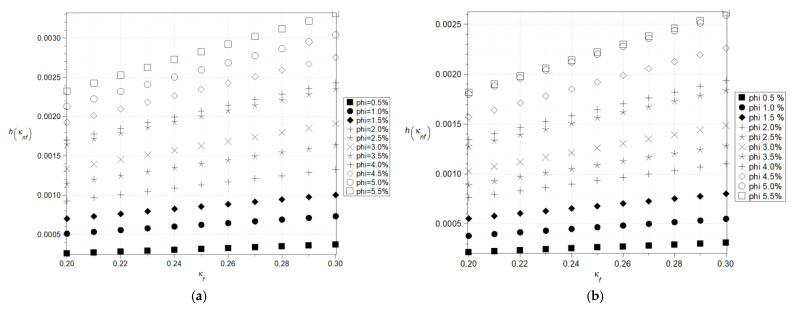
Shannon entropies for the effective thermal conductivity of the nanofluid with Al_2_O_3_ (**a**) and Cu (**b**) nanoparticles (the thermal conductivity of the base fluid varies (0.2–0.3) W/m·K).

Further, Figure 6a,b and Figure 7a,b show an analogous set of the results, the basic statistical characteristics of the effective heat conductivity. The expected values now exhibit a linear increase, closely resembling the trends observed for the effective density shown in Figure 2a. This behavior is accompanied by the thermal conductivity of the base fluid. However, the range of admissible values for different volumetric fractions is considerably narrower. Notably, the influence of these two parameters appears to be largely independent, underscoring the complexity of their interaction in nanofluid systems. These results are consistent with previous studies carried out using analytical methods [29,30] as well as due to an application of neural networks [31]. Coefficients of variation of this effective conductivity do not change while increasing input mean conductivity of the fluid, but remarkably increase while increasing the parameter *φ*. As these coefficients are constant and expectations linearly increase, one expects Shannon entropy to proportionally increase together with input fluid conductivity. This is noticeable in Figure 7a,b, where additionally, one notices expectedly that the larger the volumetric ratio of the nanoparticles, the higher the resulting entropy. However, Shannon entropy is remarkably smaller here, so uncertainty in effective heat conductivity is smaller than for effective density and heat capacity. Noteworthy is the entropy behavior observed under a significant disparity between the thermal conductivity of the base fluid and that of the nanoparticles (Figure 6b). When the volume fraction exceeds *φ* = 5%, a slight increase in entropy can be observed. This may indicate the onset of dominant interactions between nanoparticles within the nanofluid. Cu nanoparticles have a significantly smaller active surface area compared to Al_2_O_3_ nanoparticles, which enhances their thermal conductivity. This effect is reflected in the observed increase in Shannon entropy (Figure 6a,b). However, it should be noted that considering the time evolution of the system could lead to opposite conclusions due to the higher susceptibility of Cu nanoparticles to oxidation or percolation effects, which would contribute to an increase in entropy.

**Figure 8:**
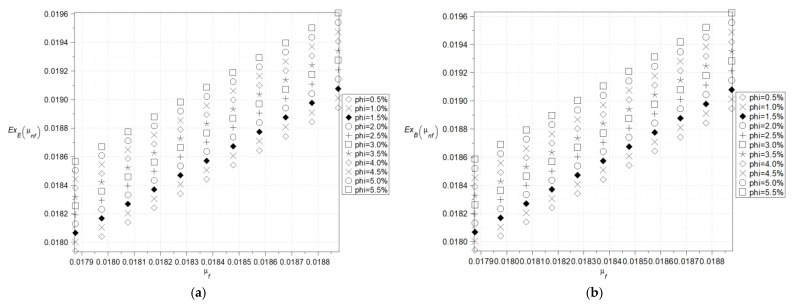
Expected values of the effective viscosity of the nanofluid in Einstein’s model (**a**), and in Brinkman’s model (**b**).

**Figure 9:**
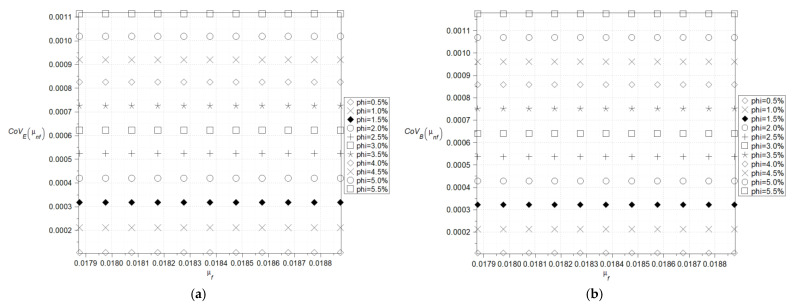
Coefficients of variation of the effective viscosity of the nanofluid in Einstein’s model (**a**), and also in Brinkman’s model (**b**).

**Figure 10:**
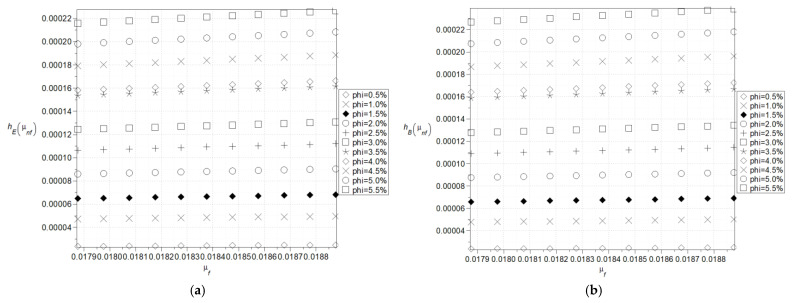
Shannon entropies for the effective viscosity of the nanofluid in Einstein’s model (**a**) and Brinkman’s model (**b**) for Al_2_O_3_.

**Figure 11:**
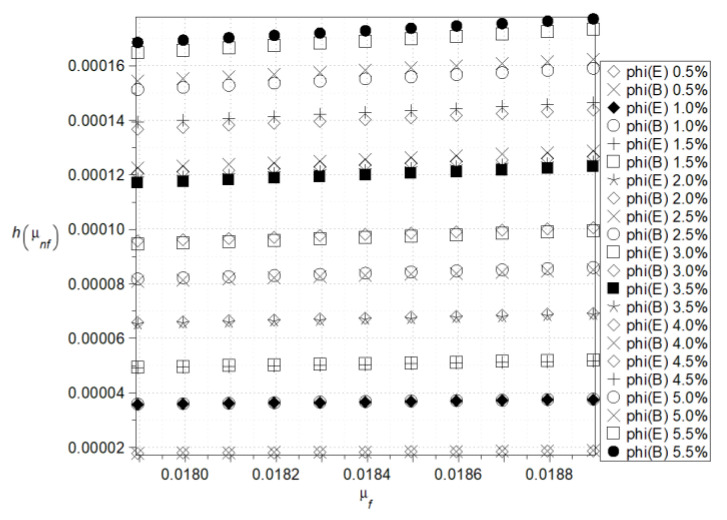
Shannon entropies for the effective viscosity of the nanofluid in Einstein’s and Brinkman’s model for Cu—comparison.

The last effective parameter under consideration is the effective viscosity calculated here in the context of both Einstein and Brinkmann models specified in the previous section; the corresponding expectations, coefficients of variations, and Shannon entropies are presented in Figure 8, Figure 9, Figure 10 and Figure 11. The left series is adjacent to the Einstein theory, whereas the right column corresponds to the Brinkmann idea. The first and most important observation is that, for the given nanofluid, these two models return almost the same statistical parameters. Expected values of the effective viscosity increase linearly together with the fluid base viscosity; it is observed for relatively small variations in the latter. Additionally, the larger the volume fraction of nanoparticles, the larger the resulting effective viscosity. The coefficients of variation (cf. Figure 9) are insensitive to any variations in the fluid base viscosity but still increase in the function of the parameter *φ*. It is noticeable that by changing this parameter from 0.5% up to 5.5%, one can increase this CoV more than five times. As a result of the expectations and coefficients of variation, one notices that Shannon entropy increases moderately while increasing input fluid viscosity, and also, remarkably, when increasing the nanoparticles’ volume ratio. Based on Figure 11, it can be concluded that the Brinkman’s model exhibits a higher entropy value than the Einstein’s model under the same input conditions. This phenomenon is clearly visible only for large values of the phi coefficient. The larger size of Al_2_O_3_ nanoparticles may initially result in a higher Shannon entropy of the effective viscosity of the nanofluid compared to Cu nanoparticles, even at relatively low volume fractions (*φ*) (Figure 10a,b and Figure 11). The greater entropy increase at high concentrations (*φ* > 1%), observed in both models for aluminum oxide, may be associated with its tendency to aggregate, indicating a potential loss of stability in the designed nanofluid parameters.

In summary, the uncertainty in the effective physical parameters of nanofluids is relatively small, typically calculated for nanoparticle volumetric ratios not exceeding 5% and their coefficient of variation (CoV) limited to 5%. However, it is important to consider that practical applications of nanofluids may require larger CoVs and could also involve an uncontrolled increase in uncertainty when the effects of temperature are taken into account. A key finding of this study is that the homogenization procedure evaluated herein does not amplify the uncertainty inherent in the enrichment of base fluids with nanoparticles. Therefore, this procedure can be efficiently applied in multiscale modeling of nanofluid flows using the Navier–Stokes equations, even in the presence of certain physical uncertainties, by common usage with the polynomial chaos approach [32], stochastic collocation method [33], stochastic perturbation method [19], and even neural network-based algorithms [34].

## 4. Concluding Remarks

A probabilistic numerical approach to the determination of the effective parameters of the nanofluids exhibiting some uncertainty in the volumetric ratio of the nanoparticles has been presented in this paper. It is based upon temperature-independent analytical formulas following some previous experimental works, Monte-Carlo simulation of Shannon entropy, and also the first two probabilistic characteristics of effective heat capacity and conductivity, mass density as well as viscosity. It has been demonstrated that Shannon entropy may serve as a single and universal uncertainty measure while analyzing statistical scattering of effective parameters contrary to multiple functions of positional statistics. This hypothesis needs to be validated for other physical problems in nanofluids, and also with larger input uncertainty levels. It is also seen that random fluctuations in effective parameters resulting from the considered uncertainty in particles’ volume ratio have rather limited values, which enables further application of the stochastic perturbation method while solving NS Equations (17)–(19) in nanofluidics for the coupled heat and mass transfer. Such an approach can be relatively easily implemented in any computer algebra system or any programming language having a statistical library together with symbolic differentiation and integration tools.

The numerical simulation reported in this paper shows that the expected value of the volumetric ratio of the nanoparticles has a noticeable impact on the resulting uncertainty of all effective physical characteristics of the given nanofluid, as detailed in [22]. This impact is many times larger than the importance of the same fluid base physical parameters. Noticeably, further increases in input uncertainty may lead to nonlinear Shannon entropy fluctuations, consistent with the physical model, and they arise when the relationship between input uncertainty and the effective properties of a nanofluid becomes increasingly complex, deviating from a straightforward proportionality. These fluctuations are consistent with the underlying physical models accounting for the nonlinearity in particle–fluid interactions and the dynamic redistribution of nanoparticles. In the context of nanofluids, the thermal and rheological properties depend heavily on nanoparticle concentration *φ*, size, shape, and distribution. As the Gaussian input uncertainty increases, its influence on the system’s effective parameters changes from linear to nonlinear behavior. This is caused due to multiple factors, such as the aggregation of nanoparticles, interparticle forces, and thermal interactions between the nanoparticles and the base fluid. These effects are captured in the variations in Shannon entropy, which encapsulates the probabilistic representation of system uncertainty, as demonstrated in this study through the analysis of effective density, specific heat capacity, thermal conductivity, and viscosity. The nonlinear entropy fluctuations highlight critical thresholds where small changes in input uncertainty lead to disproportionately large variations in effective properties. These thresholds may correspond to physical phenomena such as percolation thresholds (Cu-doped nanofluids), nanoparticle clustering (Al_2_O_3_-based nanofluids), or modifications in heat transfer pathways when particle–particle interactions become dominant.

Future work should focus on validating these findings across broader parameter ranges, incorporating temperature sensitivity, and comparing results with experimental data to ensure consistency with real nanofluid behavior. It would be interesting with no doubt to see a comparison of the NS equations’ solutions having practical importance to contrast realistic fluctuations of the nanofluid coupled behavior with their homogenized counterpart. An important direction would be an extension of the algebraic formulas for effective physical characteristics towards their temperature sensitivity [35,36] as well as applications of the nanofluidics in cellular structure analysis and biomechanics [37].

## Data Availability

Data is contained within the article.
